# Look Who Is Talking: Extracellular Vesicles as Mediators of Intercellular and Interorgan Communication

**DOI:** 10.1002/cph4.70128

**Published:** 2026-04-07

**Authors:** Michela Saviana, Giulia Romano, Daniel del Valle‐Morales, Mario Acunzo, Patrick Nana‐Sinkam

**Affiliations:** ^1^ Division of Pulmonary Diseases and Critical Care Medicine, Department of Internal Medicine Virginia Commonwealth University Richmond Virginia USA; ^2^ Department of Medicine and Surgery LUM University Casamassima Italy; ^3^ Wright Regional Center for Clinical and Translational Science Virginia Commonwealth University Richmond Virginia USA

**Keywords:** cell‐to‐cell communication, extracellular vesicles, interorgan communication

## Abstract

Extracellular vesicles (EVs) are produced by every organ, serving as vehicles for communication. By circulating throughout the body and targeting both neighboring and distant cells and organs, they can drive downstream signaling. EVs play a role as effectors of cell‐to‐cell communication in the tissue microenvironment and have demonstrated their importance in driving downstream biological processes, maintenance of homeostasis and response to stimuli. New methodologies supporting the study of EV‐mediated long‐distance communication have revealed a whole new unexplored function in maintaining homeostasis of the organism and serving as indicators of pathological conditions. In this review, we provide the most recent update on the role of EVs as mediators of intercellular and interorgan communication.

## Graphical abstract

Extracellular vesicles (EVs) mediate intercellular and interorgan communication during both physiological and pathological conditions. EVs include exosomes, ectosomes, and apoptotic bodies and contain bioactive molecules. EVs are delivered to proximal cells or can travel in bodily fluids and reach distant organs, where they trigger a response from the recipient cell.

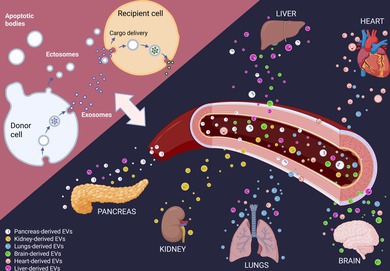

## 1 Introduction

In complex multicellular organisms, the interplay and cooperation between organs are required for development, maintenance, and repair (Droujinine and Perrimon 2013; Sun and Poss 2023; Alvarez‐Ochoa et al. 2021). The response to stimuli, maintenance of homeostasis, and adaptation are necessary for the correct function of the organism and are assured by refined cell‐to‐cell crosstalk in both short distances, between cells in the same microenvironment, and long distances between different organs. This type of signal is not only fundamental in physiological conditions and aging, but also in pathological conditions, such as metabolic syndromes, autoimmune disease, dementia, and cancers, in which organs communicate with each other to signal alterations in normal functioning (Sun and Poss 2023; Tokizane and Imai 2025; Carata et al. 2025). Such intercellular and interorgan communication is mediated by endocrine or paracrine factors, and extracellular vesicles (EVs) released by donor cells, thus reaching neighbor cells or distant organs through circulation. EVs are membrane‐coated vesicles secreted by all cells and commonly found in the extracellular environment. EVs were first identified in the 1960s (Wolf 1967; Anderson 1969), and more definitively described in the 1980s (Pan and Johnstone 1983; Johnstone et al. 1987). Aaronson and colleagues coined the term “extracellular vesicles” in reference to vesicles harboring a lipid bilayer membrane (Aaronson et al. 1971), leading in 2011 to the official use of the term and formation of the International Society of Extracellular Vesicles. EVs were initially proposed as carriers of debris in which there was an accumulation of excess material in the cell, and a mechanism to recycle or eliminate unnecessary proteins (Pan and Johnstone 1983; Harding et al. 1983; Pan et al. 1985). However, numerous lines of investigation have identified a much more essential function of EVs as key players in intercellular and interorgan communication (Zhang and Grizzle 2014; Raposo et al. 1996). EVs are constitutively released by every cell, and their cargo often reflects the content of the donor cell. A novel mechanism of genetic exchange between cells was proposed by Valadi and colleagues, who discovered that mRNA and microRNA (miRs/miRNAs) were packaged into EVs from a donor cell and delivered to another cell. These RNAs were completely functional and regulated biology in this new location (Valadi et al. 2007). On a more systemic level, once released into circulation, such vesicles can potentially reach and target any organ (Caby et al. 2005) and transport a variety of different cargo, including nucleic acids, proteins, and various metabolites, emphasizing their role in interorgan communication as well (van Niel et al. 2018).

EVs are divided based on their biogenesis into exosomes (less than 200 nm), ectosomes (100–1000 nm), and apoptotic bodies (50–5000 nm) (Jeppesen et al. 2023; Kakarla et al. 2020). The overlapping size of these vesicles makes any approach to discrimination difficult, such that EVs can be classified as small EVs (generally less than 200 nm) and larger EVs (Welsh et al. 2024). The presence of specific surface markers and content can facilitate the distinction between different types of small EVs: ALG‐interacting protein X, TSG101, and the tetraspanins (four transmembrane domain‐containing proteins) CD9, CD63, and CD81 have been recommended as exosome markers in the past; however, their presence is not exosome‐specific and appears to also be present in other EVs (Crescitelli et al. 2013; Mathieu et al. 2021; Kowal et al. 2016). To date, there are also no universal molecular markers of EVs or EV subtypes. Based on the most current Minimal Information for Studies of Extracellular Vesicles (MISEV) guidelines, the most correct discrimination of EVs is based on their biogenesis, and, unless their biogenesis can be demonstrated, it is not recommended to discriminate between different types of EVs (Welsh et al. 2024). Given the current technical limitations in definitively isolating and characterizing pure exosomes, we chose to adopt the broader term “extracellular vesicles (EVs)” throughout this review. This approach aligns with the recommendations from MISEV2023 and allows for a more inclusive discussion of the literature, especially when the original studies may not fully adhere to established EV characterization guidelines.

EV content can be driven by their subtype, cells of origin, and physiological conditions (Abels and Breakefield 2016). In this regard, several publicly available databases (Exocarta, Vesiclepedia, and EVpedia) have been assembled from hundreds of studies (Kim et al. 2013; Kalra et al. 2012; Keerthikumar et al. 2016). These vesicles can deliver their cargo and effectively alter the biological response of target cells in both physiological and pathological conditions. As such, EVs are associated with maintaining homeostasis, reproductive function, aging, and disease response. Interestingly, EVs can cross both the blood–brain (Banks et al. 2020) and maternal‐fetal barriers (Sheller‐Miller et al. 2019) and be artificially engineered to deliver therapeutic cargoes, such as chemotherapeutic agents, shRNA, and immune modulators. Moreover, given their identification in all biological fluids, they may serve as diagnostic, prognostic, and therapeutic biomarkers.

In this review, we will focus on the role of EVs as mediators of biological communication, providing a general view from cell‐to‐cell interaction to long‐distance communication between different organs.

## 2 Biology of Extracellular Vesicles

**FIGURE 1:**
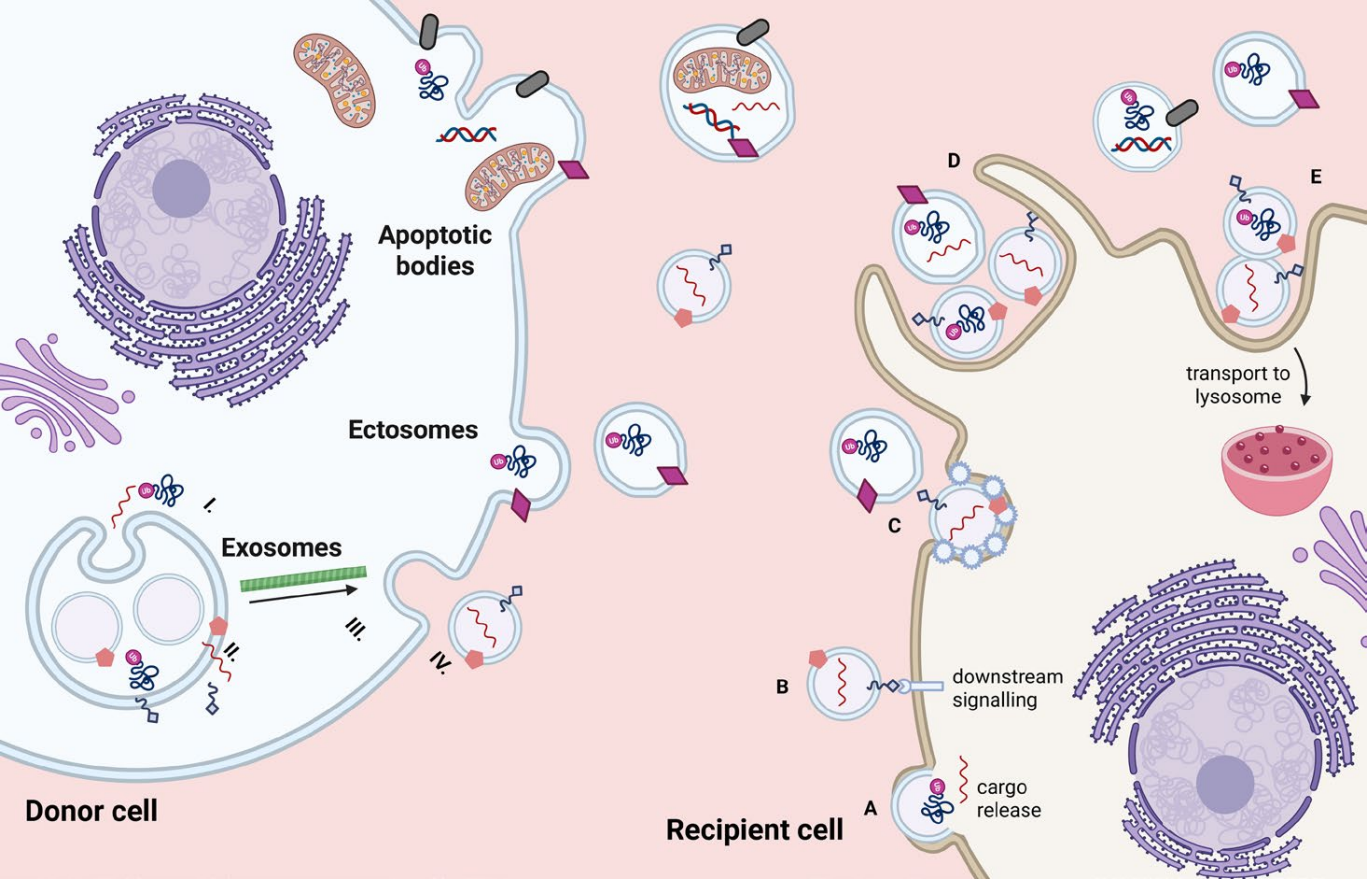
Schematic representation of extracellular vesicles (EVs) biogenesis and uptake. Apoptotic bodies (50–5000 nm) originate from the blebbing of the plasma membrane of apoptotic cells. Ectosomes (100–1000 nm) are produced when lipids and cytoskeletal proteins reorganize and determine budding of the plasma membrane. Exosome biogenesis involves the cargo selection of biomolecules such as lipids, proteins, DNA, and RNAs to the multivesicular bodies (MVB) (I). Once the cargo has been recruited, intraluminal vesicles (ILVs) are formed through the inward budding of the endosomal membrane by either the endosomal sorting complex required for the transport (ESCRT)‐dependent or the ESCRT‐independent pathway (II). Once matured, the MVB is transported to the plasma membrane by the cytoskeleton (III). Fusion of the MVBs to the plasma membrane occurs through the Rab GTPase family, SNARE complex, and tethering proteins, releasing the exosomes (IV). The EVs are uptaken by fusion with the plasma membrane of the recipient cell (A) or they can directly interact with surface receptors in the plasma membrane and trigger the signaling cascade (B). EVs can also be internalized through endocytosis mediated by specific transmembrane proteins, lipids, or ligands (C). EVs can also be uptaken and recycled by macropinocytosis (D), a process to internalize EV, which will then fuse with late endosomes or lysosomes, or by phagocytosis (E), which directs EVs cargo to the lysosomes. Created in https://BioRender.com.

Cells can produce a wide range of EVs classified based on their biogenesis (Figure 1). Here, we summarize the biogenesis of exosomes, ectosomes, and apoptotic bodies and the mechanisms of uptake.

### 2.1 Exosomes Biogenesis

Exosome biogenesis and export consist of a series of orchestrated steps: cargo selection and recruitment to the multivesicular body (MVB), MVB maturation, MVB transport to the plasma membrane, and MVB fusion with the plasma membrane, releasing exosomes. The main mechanism for intraluminal vesicles (ILVs) formation is the endosomal sorting complex required for the transport (ESCRT) pathway, although other ESCRT‐independent pathways have been described in the literature (Han et al. 2022). Sorting of biomolecules such as lipids, proteins, DNA, and RNA into exosomes relies on either the ESCRT‐dependent or ‐independent pathways.

#### 2.1.1 ESCRT Dependent Pathways

The ESCRT complex comprises five core complexes that cooperate in stepwise fashion: ESCRT‐0, I, II, III, and vacuolar protein sorting 4 (VPS4) (Schmidt and Teis 2012). The ESCRT mechanism is initiated with the binding of ESCRT‐0 to ubiquitinated proteins. ESCRT‐0 then binds to lipid phosphatidylinositol 3‐phosphate (PI3P) of the endosome to capture the cargo. ESCRT‐I and ESCRT‐II are recruited to the complex, which forms a saddle‐shaped complex to facilitate the assembly of ESCRT‐III (Schöneberg et al. 2017). ESCRT‐III is polymerized in the complex through the energy provided by ATP hydrolysis of VPS4 (Stuchell‐Brereton et al. 2007), promoting membrane deformation and budding (Pfitzner et al. 2020). This budding results in the formation of ILVs. The ESCRT complex can recruit deubiquitinating enzymes to the ILVs as well, but it is not a requirement for all cargo proteins (Huebner et al. 2016).

Several other proteins can recruit and initiate the ESCRT pathway to form ILVs. Alix is well described to recruit and nucleate ESCRT‐III. The Syndecan‐Syntenin‐Alix pathway can form ILVs by cargo recognition, such as recognition of Fibroblast Growth Factor Receptor (FGFR) and lysyl‐tRNA synthetase (Kim et al. 2017; Roucourt et al. 2015). Integrins can directly recruit ESCRT‐0, which recruits HD‐PTP (Kazan et al. 2021). HD‐PTP recruits ESCRT‐III and VPS4 to form the ILVs. These ILVs are stored and sent to the lysosome for degradation (Wenzel et al. 2018). A component of ESCRT‐I, Tsg101 can recognize galectin‐3 and BAG6 to initiate the ESCRT‐dependent pathway (Schuldner et al. 2019; Bänfer et al. 2018).

Proteins are sorted into exosomes through mono‐ubiquitination and recognition by ESCRT‐0 as described above. RNAs are loaded into exosomes by sequence recognition of RNA‐binding proteins. Several classes of RNA, including mRNAs (Tomasoni et al. 2013), noncoding RNAs (ncRNAs) (Qiu et al. 2021), miRNAs (Villarroya‐Beltri et al. 2013), and circular RNAs (circRNAs) (Pan et al. 2022), may serve as cargo in exosomes. miRNAs are the most well‐described cargo for exosomes; the RNA‐binding protein hnRNPAB1 loads miRNAs into exosomes (Siculella et al. 2023). miRNAs were identified to have a common motif sequence in which hnRNPA2B1 can recognize and sort miRNAs (Villarroya‐Beltri et al. 2013). The RNA‐induced silencing complex component Ago2 has been reported to be sorted into exosomes and is responsible for sorting specific miRNAs, including let‐7a, miR‐100, and miR‐320a (McKenzie et al. 2016). Interestingly, the RNA modification N6‐methyladenosine has been reported to inhibit the AGO2/miRNA interaction and promote recognition of hnRNPA2B1, promoting the loading of miRNAs to exosomes (Garbo et al. 2024).

#### 2.1.2 ESCRT Independent Pathways

The lipid composition and associated proteins of the MVB membrane drive ESCRT‐independent formation of ILVs. Sphingolipids, cholesterol, phosphatidylserine, membrane proteins, and ceramides form structures similar to lipid rafts and induce the budding of MVBs into ILVs (Ghadami and Dellinger 2023). Neutral sphingomyelinase 2 (nSMase2) converts sphingomyelin to ceramide; ceramide can induce budding and formation of ILVs (Trajkovic et al. 2008). Knockdown of nSMase2 reduces ILVs of certain cargoes (Trajkovic et al. 2008). EFGR can phosphorylate RAB31 to promote RAB31 interaction with flotillin proteins in lipid rafts and drive EFGR entry into ILVs (Wei et al. 2021). Tetraspanins such as TSPN6 (Ghossoub et al. 2020), and CD63 (van Niel et al. 2011) can sort and promote ILV formation (Andreu and Yáñez‐Mó 2014). The lysosome‐associated membrane protein 2, isoform A (LAMP2A) sorts proteins with a KFERQ‐like motif into exosomes through interactions with CD63, Alix, Syntenin‐1, and RAB31 (Ferreira, da Rosa Soares, et al. 2022).

Exosomes carry lipid cargo either within the exosome or on the outer membrane (Donoso‐Quezada et al. 2021). As mentioned earlier, the membrane of exosomes can carry cholesterol, ceramides, sphingomyelin, and phosphatidylserine. For example, hepatocytes release exosomes enriched in ceramides that induce a pro‐inflammatory response (Kakazu et al. 2016). Adipocytes secrete lipid‐filled exosomes that are taken up by macrophages (Flaherty 3rd et al. 2019). The distribution of membrane lipids can vary by exosome. For example, phosphatidylserine is typically found in the inner membrane of the exosome and is externalized in apoptotic or malignant cells to promote their uptake by macrophages (Sharma et al. 2017).

MVBs are transported to the plasma membrane through the interaction of the cytoskeleton, actin, and microtubules (Villarroya‐Beltri et al. 2014). The actin‐binding protein Cortactin directs the trafficking and docking to the plasma membrane (Sinha et al. 2016). Once near the vicinity of the plasma membrane, members of the Rab GTPase family, SNARE complex, and tethering proteins contribute to the fusion of MVBs (Krylova and Feng 2023). Rab27a, Rab27b, and Rab35 recruit specific tethers to V‐SNARE found in MVBs and T‐SNARE found in the plasma membrane (Pfeffer 2007; Bröcker et al. 2010). The exosomes are released once the MVB docks and fuses with the plasma membrane.

### 2.2 Ectosomes Biogenesis

Ectosomes originate from outward budding of the plasma membrane, determined by a reorganization of membrane lipids and cytoskeletal proteins (Tricarico et al. 2016). Briefly, alterations to the plasma membrane, lipid composition, and membrane instability drive the formation of microdomains where the budding will occur. Proteins, nucleic acids, lipids, and metabolites are sorted and recruited to the bud site. Once the budding of the ectosome is formed, in some contexts ESCRT‐III performs the scission, and the ectosome is released into the extracellular space. Several mechanisms drive these biophysical deformities including mechanisms shared with exosomes such as the ESCRT proteins (Tricarico et al. 2016). The arrestin domain‐containing protein 1 (ARRDC) can recruit and localize ESCRT‐I protein TSG101 to the plasma membrane, where it drives budding and ectosome formation (Nabhan et al. 2012). Cellular stimuli can increase the concentration of Ca^2+^ and recruit calcium‐dependent enzymes, that, with altered flippases and floppases activity, alter membrane composition for budding (Yu, Sane, et al. 2024; Taylor et al. 2020).

### 2.3 Apoptotic Bodies Biogenesis

Apoptotic bodies are produced during the regulated dismantling of apoptotic cells. Such vesicles largely differ in size and cargo, as they contain organelles like mitochondria, reticulum, and fragments of the nucleus (Gregory and Rimmer 2023). The formation of apoptotic bodies occurs via blebbing of the plasma membrane into the formation of vesicles of different sizes. This process is induced by Rho‐associated coiled‐coil‐containing protein kinase 1 (ROCK1), which is activated by cleaved caspase 3 during the apoptotic cascade (Coleman et al. 2001; Sebbagh et al. 2001). ROCK1 activates myosin light chain (MLC) and drives actomyosin contractility. Membrane blebs start similarly to the formation of protrusions that allow cell migration, with subsequent growth and detachment of apoptotic bodies (Gregory and Rimmer 2023). Apoptotic cells flip the phosphatidylserine from the inner membrane to the outer membrane, inducing a “eat me” signal to macrophages (Kim et al. 2022).

### 2.4 EV Uptake

EVs secreted from parental cells can interact with recipient cells by three different mechanisms (Gurung et al. 2021):

(1) Fusion with the plasma membrane: The lipid bilayer of EVs fuses with the lipid bilayer of the plasma membrane, allowing the release of EV content directly inside the recipient cell. SNAREs and Rab protein families, integrins, adhesion molecules, and lipid raft‐like domains mediate this process (Chernomordik et al. 1987; Prada and Meldolesi 2016; Valapala and Vishwanatha 2011; Mulcahy et al. 2014).

(2) Direct contact: EVs can trigger the signal cascade in the recipient cells by direct contact through surface receptors. EVs involved in immunomodulatory and apoptotic functions generally express surface molecules that interact with the receptors on the plasma membrane of the recipient cell, inducing its activation (Munich et al. 2012; Sobo‐Vujanovic et al. 2014; Guan et al. 2014).

(3) Internalization: EVs are internalized by recipient cells, where they fuse with the intracellular compartments for subsequent cargo release. Internalization can occur in multiple ways, including endocytosis, phagocytosis, or macropinocytosis. In Clathrin‐mediated endocytosis, various transmembrane receptors and ligands are assembled in the plasma membrane to create the endocytic site. The cargo is recruited to this site, and the membrane shaped into an invagination, which is then separated from the plasma membrane (Kaksonen and Roux 2018). The vesicles are finally uncoated and fuse with the endosomes. Similarly, Caveolins, integral membrane proteins, create small, coated invaginations of the membrane, facilitating Caveolin‐mediated endocytosis of EVs (Kiss and Botos 2009). Endocytosis can also be mediated by lipid rafts, portions of the plasma membrane enriched in cholesterol and sphingolipids that facilitate EVs internalization. During EVs phagocytosis, the cell membrane deforms and encircles extracellular particles, forming phagosomes, which direct their cargo to lysosomes (Gordon 2016). Macropinocytosis occurs through lamellipodia to create inward plasma membrane invaginations that collapse back into the plasma membrane to form intracellular compartments called macropinosomes. These macropinosomes then fuse with late endosomes or lysosomes (Kerr et al. 2006; Hewlett et al. 1994). The mechanisms of EV uptake are illustrated in Figure 1.

## 3 EV‐Mediated Communication

### 3.1 Cell‐To‐Cell Communication

**FIGURE 2:**
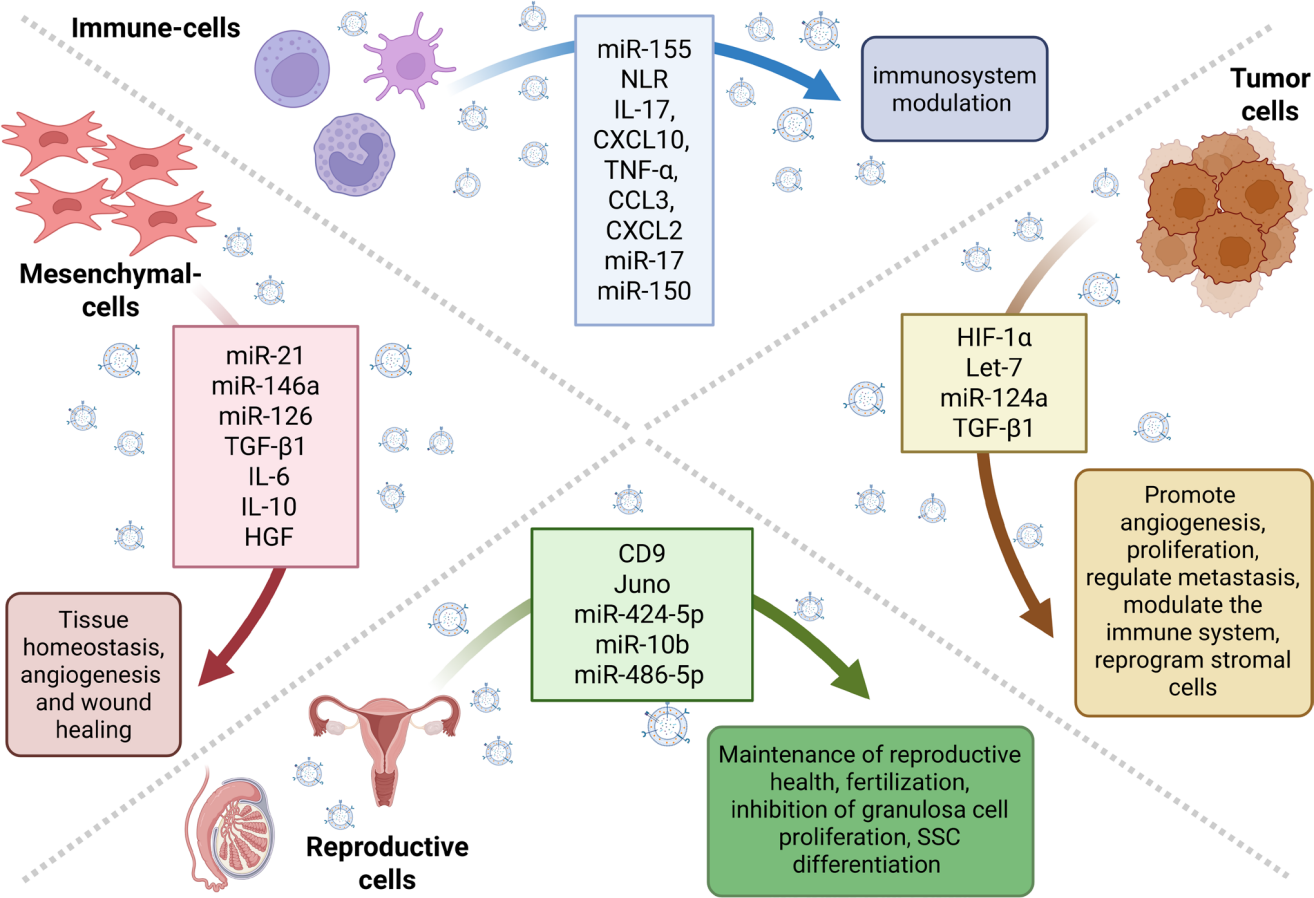
Extracellular vesicles (EVs) as mediators of cell–cell communication across diverse cell types. This figure describes the role of EVs in intercellular communication, highlighting their origins, cargo, and functional outcomes. Four major EV‐producing cell types are illustrated: Tumor cells (brown), immune cells (blue), mesenchymal cells (red), and reproductive cells (green), each shown releasing EVs toward their respective targets. Arrows indicate the direction of communication, while boxes summarize the molecular cargo contained within the EVs and the downstream biological effects they mediate. Tumor cells (brown): Tumor‐derived EVs are enriched in oncogenic miRNAs (e.g., Let‐7 and miR‐124a) and proteins (e.g., transforming growth factor beta (TGF‐β1), hypoxia inducible factor 1 alpha (HIF‐1α)) that promote tumor progression. These EVs contribute to extracellular matrix (ECM) remodeling, enhance metastatic potential, and suppress anti‐tumor immune responses, thereby shaping a tumor‐supportive microenvironment. Immune cells (blue): EVs from immune cells, including dendritic cells, macrophages, and T lymphocytes, contain immunoregulatory molecules (e.g., CXC motif chemokine ligand 10 (CXCL10), tumor necrosis factor alpha (TNF‐α), CXC motif chemokine ligand 2 (CXCL2), Nod‐like receptor (NLR), CC motif chemokine ligand 3 (CCL3), and Interleukin‐17), and immune‐related miRNAs (e.g., miR‐155, miR‐150, and miR‐17). These vesicles regulate antigen presentation, inflammation, and the activation of immune cells. Mesenchymal cells (red): Mesenchymal stromal cell (MSC)‐derived EVs carry miRNAs (e.g., miR‐21, miR‐126, and miR‐146a) and proteins (TGF‐β1, Interleukins 6 and 10, and Hepatocyte growth factor (HGF)) that influence ECM organization, modulate fibroblast activity, and promote tissue repair. These vesicles are essential for maintaining tissue homeostasis, regulating angiogenesis, and wound healing. Reproductive cells (green): EVs secreted by reproductive cells (e.g., oocytes, Sertoli cells, and granulosa cells) carry reproductive‐specific cargo, including the tetraspanin CD9, Juno, miR‐424‐5p, miR‐486‐5p, and miR‐10. These EVs contribute to gamete maturation, fertilization, and signaling in the reproductive tract. They also play roles in inhibiting granulosa cell proliferation and promoting spermatogonial stem cell (SSC) differentiation, thereby ensuring reproductive competence. Created in https://BioRender.com.

Cell–cell communication is an essential biological feature in responding to stimuli in stress or natural conditions and ensuring the maintenance of homeostasis (Figure 2).

EVs are present and stable in many body fluids (blood, amniotic fluid, urine, milk, and cerebrospinal fluid) (Martins et al. 2023; Drusco et al. 2015; Kumar et al. 2024). A central role for EVs is in mediating intercellular communication, both locally and over long distances, by transferring their molecular cargo between cells (Zeng et al. 2023). While almost every cell type can release EVs, specific cells are known to release a higher quantity of EVs due to their role in cell‐to‐cell communication, primarily involving immune responses, endocrine communication, and tissue maintenance. Among others, EVs derived from immune cells, including dendritic cells, T‐cells, and macrophages, can modulate the immune response (dendritic cells), facilitate communication between immune cells (T‐cells), and influence inflammation (macrophages) (Hsu et al. 2024; Kwantwi 2023).

Beyond immune cells, tumor cells release significant quantities of EVs, contributing to cell‐to‐cell interactions, influencing tumor progression, metastasis, and immune evasion (Hsu et al. 2024; Kwantwi 2023). Stem cell‐derived EVs enriched in protein, lipids, and RNA play an essential role in tissue regeneration and repair (Zhou, Zhang, et al. 2023; Tan et al. 2024). Additionally, platelet‐derived EVs have been shown to induce coagulation, wound healing, and communication between platelets and other cell types (Wei et al. 2022; Aatonen et al. 2012). Vascular endothelial cells release EVs to maintain vascular homeostasis and regulate immune cell recruitment in their environment (Zeng et al. 2021). Finally, neurons and glial cells release EVs, which play roles in neurodegenerative diseases and cell‐to‐cell communication within the brain (Oyarce et al. 2022).

Collectively, the functional diversity of EVs reflects the specialized roles of their cell origin, highlighting their significance in both physiological and pathological processes. All cells are potential targets for EV signaling. However, their ability to respond to EVs signaling depends on the presence of specific receptors and/or active pathways that can interact with bioactive molecules carried within EVs. Proteins, lipids, and RNA in EVs can all interact with target cells to influence various cellular processes, such as (1) Signal transduction, (2) Gene regulation, and (3) Immune modulation.

By delivering their cargo, EVs can impact recipient cell biology in beneficial or harmful ways. They are the foremost mediators of intercellular communication in short and long‐range processes such as immune system regulation, nervous system and endocrine signaling, tissue homeostasis and repair, neuronal communication, metabolic and hormonal regulation, and cancer surveillance and prevention. Such an essential role in intercellular communication positions them to be involved in several disease states, including cardiovascular disease, diabetes mellitus, prion dissemination, inflammation, viral infection, and tumorigenesis (Hushmandi et al. 2024). In this section, we focus on EV‐mediated communication occurring within a single tissue or endocrine microenvironment, where both EV‐producing and EV‐recipient cells reside locally; we discuss the most relevant intercellular signaling mediated by EVs released from the immune cells, mesenchymal cells, tumors, and endocrine cells (summarized in Figure 2). Many of these signals involve the transport of EV‐encapsulated miRNA, summarized in Table 1.

**TABLE 1:** Summary of the cited miRNAs involved in intercellular and interorgan communication mediated by EVs.

Source tissue/cell	miRNA cargo	Recipient cell/tissue	Effect	References
Dendritic cells	miR‐155	T cells	Stimulates T cell activation	Alexander et al. (2015); Montecalvo et al. (2012)
Macrophages	miR‐155	Cardiac fibroblast	Increases inflammation	Wang et al. (2017)
Activated lymphocytes	miR‐150	N/A	miR‐150 is secreted in EVs to downmodulate its levels for lymphocyte differentiation	de Candia et al. (2013)
Gastric cancer cells	Let‐7	N/A	Secreted into the extracellular space to downregulate Let‐7 and promote oncogenesis	Ohshima et al. (2010)
Pancreatic islet β‐cells	miR‐1224, miR‐133a, miR‐423‐5p	Naïve β‐cells	Impairs glucose‐stimulated insulin secretion	Yu, Yang, et al. (2024)
Pancreatic β‐cells	miR‐127	Endothelial pancreatic cells	Increased pancreatic tube formation	Shen et al. (2019)
Pancreatic stellate cells	MiR‐23a‐3p	β‐cells	Released during hypoxia and induces apoptosis	Lee et al. (2023)
Antral follicle	miR‐204, miR‐92b, miR‐328a‐3p, miR‐424‐3p, miR‐450a	Antral follicle	Shift in miR EV profile promotes the maturation of the antral follicle	Navakanitworakul et al. (2016)
Antral follicle	miR‐424‐5p	Antral follicle	In polycystic ovary syndrome, EV miR‐424‐5p is downregulated. EV miR‐424‐5p inhibits cell proliferation	Yuan et al. (2021)
Sertoli cells	miR‐10b	Primary spermatogonia	Inhibits apoptosis by targeting KLF4	Gao et al. (2023)
Sertoli cells	miR‐486‐5p	Primary spermatogonia	Facilitates differentiation by downregulating PTEN	Gao et al. (2023)
Bone marrow mesenchymal stem cells	miR‐124	Brain	Ameliorates stroke‐mediated injury and reduces inflammation	Tian, Yao, et al. (2022)
Bone marrow mesenchymal stem cells	miR‐23a‐3p	Brain	Polarization to M2 phenotype	Dong et al. (2022)
Liver	miR‐3075	Adipocytes	Sensitizes the cell to insulin	Ji et al. (2021)
Adipocyte	miR‐99b	Liver	Inhibits FGF21 to improve glucose tolerance	Thomou et al. (2017)
Adipocyte	miR‐141‐3p	Liver	Obese adipocytes release less miR‐141‐3p, which inhibits insulin sensitivity	Dang et al. (2019)
Heart	miR‐23‐27‐24	Adipocyte	Downregulates EDEM3, which induces endoplasmic reticulum stress	Gan et al. (2022)
Heart	miR‐221, miR‐21, miR‐24, miR‐214	Lung	Accelerates tumor growth in mice and activates pro‐tumorigenic macrophages	Caller et al. (2024)
Pancreas	miR‐223	Liver, skeletal muscle	Facilitates glucose uptake	Wei et al. (2023); Zhang et al. (2018)
Pancreas	miR‐29s	Liver	Modulates insulin sensitivity	Li et al. (2021)
Pancreas	miR‐26a	Liver	Downregulation of EV miR‐26a induces insulin resistance	Xu et al. (2020)

#### 3.1.1 Immune Cell‐Derived EVs

Immune system cells‐derived EVs represent the best example of mediators of cell‐to‐cell communication based on action through both paracrine and autocrine forms, creating an optimal microenvironment for immune cell function (Hazrati et al. 2022). Proteins with immune‐modulating functions, MHC‐I, MHC‐II, costimulatory proteins (CD86), and adhesion proteins (CD11b, CD54/ICAM) can be found enriched in EVs (Segura et al. 2007, 2005; Clayton et al. 2004). Some EVs carrying both MHC‐I and MHC‐II complexes are required to prime T‐Cells and can activate T‐Cell receptors (TCR) on T lymphocytes (Utsugi‐Kobukai et al. 2003). Interestingly, the presence of functional MHC–antigenic peptide complexes could support EVs‐mediated initiation of adaptive immune responses (Pelissier Vatter et al. 2021).

Dendritic cells (DC) can release several subtypes of EVs depending on the microenvironment. Dendritic cell‐derived EVs (DCEVs) can activate the innate immune system by activating specific T‐Cells (Viaud et al. 2009; Théry et al. 2002). DCEVs containing miR‐155 can influence T cell responses and participate in antigen presentation (Alexander et al. 2015; Montecalvo et al. 2012). DCs can produce IL‐10 and express Fas‐Ligand on the surface when activated in a tolerogenic environment; in this case, DCEVs can suppress autoimmune responses (Kim et al. 2006). DCEVs are unique and have been investigated as a potential therapeutic in many diseases, such as cancer immunotherapy and the recruitment of mesenchymal stem cells (MSCs) for tissue repair (Pitt et al. 2016; Silva et al. 2017).

The protein composition of macrophage‐derived EVs (MDEVs) changes following activation; levels of NOD‐like receptor (NLR) related proteins, cytokines, and chemokines such as IL‐17, CXCL10, TNF‐α, CCL3, and CXCL2 increase. Conversely, miR‐17, which negatively regulates ICAM, is reduced (Hazrati et al. 2022; Wang, Jin, et al. 2019). Furthermore, activated macrophages can release EVs, inducing an inflammatory response by activating TNF‐related signaling pathways, the NLRP3 receptor‐dependent inflammasome, and TOLL‐like receptors (TLR) (Wang, Jin, et al. 2019). MiR‐155 carried by MDEVs can reduce cell proliferation and increase inflammation in cardiac fibroblast cells. Its target, the SOS1 gene, is a master regulator of Ras activation (Wang et al. 2017). Furthermore, activated MDEVs can also increase inflammation in hepatocytes, causing acute liver injury (Wang, Jin, et al. 2019) and in vascular endothelial cells under hypertensive conditions (Osada‐Oka et al. 2017). Finally, MDEVs, through the deregulation of integrin β1 and αMβ2 integrin, can inhibit endothelial and tumor cell migration in hepatocellular carcinoma (HCC) (Lu et al. 2023).

Natural Killer (NK) cells constantly produce EVs. NK‐activated EVs can trigger the activation of quiescent NK; interestingly, Fas‐L (membrane‐bounded and soluble form) is present in all NK‐derived EVs, but it is heavier in the activated ones (50 kD instead of 40 kD) (Lugini et al. 2012). Fas‐L and perforin in NK‐activated EVs induce apoptosis in tumor cells (Zhu et al. 2017). NK EVs play a role against activated immune cells. In this way, they help maintain homeostasis of the immune response (Fais 2013). Finally, the uptake of these EVs through micropinocytosis by tumor cells can block NK cytolytic activity, blocking the CD226 on their surface (Enomoto et al. 2022).

Activated T‐cell EVs (TCEVs) can increase proliferation in resting autologous cells. Their cargo is enriched in miRNAs, and transferring these small RNAs enhances the APC antigen capability (Mittelbrunn et al. 2011). MiR‐150 is a key repressor of lymphocytes, and it has been shown that flu vaccine‐activated lymphocytes can downmodulate its level, releasing it in EVs (de Candia et al. 2013).

ERK and NF‐KB pathways in melanoma cells are activated by CD8+ TCEVs, thus, increasing their metastatic behavior (Cai et al. 2012). TCEVs can mimic CAR‐T cell receptor characteristics, and their therapeutic use has been proposed to avoid CAR‐T cell cytokine release syndrome (CRS) (hypotension, nausea, tachycardia, and headache). The advantages are mainly three: (1) they can transfer granzyme and lysosomal enzyme directly to the target without the perforin. (2) their small size allows them to cross blood–brain and tumor‐forming barriers arriving directly to the cells. (3) antitumor drugs can be loaded in these EVs (Hazrati et al. 2022).

#### 3.1.2 Mesenchymal Stem Cells‐Derived EVs

Another physiological role for EV‐mediated short‐distance communication is in driving tissue homeostasis and wound healing. A fine and accurate regulation of somatic stem activity is necessary to maintain tissue homeostasis. These cells must respond to tissue damage and proliferate according to tissue requirements while avoiding over‐proliferation (Biteau et al. 2011). Tissue injury, for example, is often characterized by tissue acidosis, and it has been shown that EVs uptake is dependent on intracellular and microenvironmental acidity, so injured tissue cells are more prone to endocytose EVs (Parolini et al. 2009; Dimitrov et al. 1990). Mesenchymal stem cells (MSCs) are stromal stem cells in many adult tissues. They can differentiate into all the embryonic lineage (ecto‐, meso‐, and endoderm) (Dominici et al. 2006) playing an essential role in tissue homeostasis, as well as in wound repair and tissue regeneration. MSCs are one of the most EVs‐secreting cell lines present in adult tissues (Nikfarjam et al. 2020). EVs secreted after pretreating MSCs with the drug pioglitazone can induce angiogenesis, activate the Akt and eNOS levels, and support wound healing (Hu et al. 2021; Qiu et al. 2020). The long non‐coding RNA (lncRNA) H19 released in adipose MSCs EVs can downregulate miR‐19b and upregulate its target SOX9 and consequently activate the Wnt/β‐Catenin pathway, enhancing fibroblast migration and proliferation, accelerating skin wound healing (Qian et al. 2021). MSCs can regulate adaptive as well as innate immune cells. Evidence suggests that MSCs exert their immunomodulatory function through the paracrine pathway, primarily via EVs (Phinney and Pittenger 2017; Liang et al. 2014). Secreted MSC EVs contain lipids, proteins, and RNA that can modify an adjacent cell's behavior. MSC‐derived EVs enriched with specific miRNA can further enhance the intrinsic ability of recipient cells to prevent apoptosis, promote angiogenesis, and induce myocardial cell proliferation (Wang et al. 2015). For example, miRNAs such as miR‐21, miR‐146a, and miR‐126, often found in MSC EVs, have been implicated in these processes (Wang et al. 2015; Song et al. 2017; Gong et al. 2017). Many investigators also report that MSC‐derived EVs contain cytokines and growth factors such as TGF‐β1, IL‐6, IL‐10, and HGF, which are essential for promoting angiogenesis and tissue repair (Yin et al. 2019). In preclinical models, MSC‐derived EVs have demonstrated therapeutic potential beyond wound healing. In letrozole‐induced mouse models of polycystic ovary syndrome (PCOS), injection of MSC EVs reversed key pathological features, including fertility impairment, hormonal imbalance, and ovarian histopathology, illustrating their regenerative and immunomodulatory capabilities (Park et al. 2023).

#### 3.1.3 Tumor Microenvironment‐Derived EVs

EVs‐mediated cell‐to‐cell communication is essential for remodeling the microenvironment and forming premetastatic niches during cancer development (Tai et al. 2018). Tumor‐derived EVs can induce autocrine/paracrine oncogenesis, promote angiogenesis, and modulate the immune system while reprogramming stromal cells (Maia et al. 2018). They can suppress cell proliferation and anticancer cytolytic functions and ablate monocyte differentiation into dendritic cells (Valenti et al. 2006). Furthermore, TGF‐β1 inside tumor EVs can activate proliferation in an autocrine manner (Raimondo et al. 2015). Investigators have demonstrated that some metabolites present in tumor microenvironments can, through a paracrine route, often be packaged in cancer‐associated fibroblast (CAF) EVs and can transform normal CAFs into a cancerous state (Zhao et al. 2016). Let‐7 plays a strategic tumor suppressor role, and cancer cells can release let‐7 packaged within EVs to promote oncogenesis (Ohshima et al. 2010). miR‐124a in adenocarcinoma cell lines can regulate EV release targeting Rab27a and Rab‐32, regulating the metastatic process (Romano et al. 2020). As mentioned earlier, proteins containing a KFERQ motif are loaded in EVs with a membrane protein LAMP2A‐dependent mechanism; the master regulator of Hypoxia HIF1A is loaded into EVs using this mechanism to transport hypoxic signaling to normoxic cells, thus activating neovascularization in vivo (Ferreira, da Rosa Soares, et al. 2022). Additionally, it has been shown that EVs derived from mesenchymal stem cells can have cancer‐suppressing effects; by reducing extravillous trophoblast apoptosis and enhancing the invasive ability of these cells, they may help repair the pathophysiology of preeclampsia (Matsubara et al. 2021).

#### 3.1.4 Endocrine Cell‐Derived EVs

The endocrine system depends on precisely regulated communication between cells and organs, traditionally mediated by hormones acting over short and long distances. Recently, the study of EVs has revealed an additional, more nuanced layer of endocrine signaling. This allows endocrine cells to refine, modulate, or fine‐tune hormonal responses beyond the classical hormone–receptor interactions.

##### 3.1.4.1 Insulin Signaling and Glucose Homeostasis

One of the most well‐studied areas of EV‐based endocrine signaling is insulin signaling and glucose regulation. β‐cell‐derived EVs can affect other β‐cells (autocrine/paracrine) in endocrine communication. Guay et al. observed a change in the miRNA cargo of β‐cell‐derived EVs when treated with pro‐inflammatory cytokines, and EVs from cytokine‐treated MIN6B1 cells induce apoptosis in the recipient MIN6B1 cells (Guay et al. 2015). An interesting study shows that pancreatic islets secrete preproinsulin on the surface of EVs that activate the insulin signaling pathway in an insulin receptor manner (Ghosh et al. 2024). In a recent paper, Yu et al. demonstrate how EVs from insulinoma β‐cells can impair glucose‐stimulated insulin secretion in recipient β‐cells by transferring miRNAs, such as miR‐1224, miR‐133a‐3p, and miR‐423‐5p, which modulate key signaling proteins (phospho‐Akt, CaMKII, and GLUT2) in recipient cells (Yu, Yang, et al. 2024). Pancreatic β‐cells also interact with other cells within the same tissue/organ in a cohesive cross‐regulation. EVs originating from β‐cells play an important role in preserving pancreatic islet function (Sun et al. 2019). EVs enriched in miR‐127 isolated from murine pancreatic β‐cells (MIN6) can be transferred to MS1 endothelial pancreatic cells, which exhibit increased tube formation ability, suggesting an effect of β‐cells in regulating endothelial cell migration and tube formation (Shen et al. 2019). Type 1 diabetes is characterized by inflammatory events in the β‐cell microenvironment, which causes immune cell activation and infiltration of the islets of Langerhans by auto‐reactive cytotoxic T‐lymphocytes. In response to exposure to pro‐inflammatory cytokines, stressed β‐cells release a high number of EVs enriched in cytokines and TLR‐binding miRNA that promote activation of macrophages and dendritic cells (Giri et al. 2020). Pancreatic stellate cells are activated during hypoxia, a condition that occurs in Type 2 diabetes mellitus. Once activated by hypoxia, these cells release EVs enriched with miR‐23a‐3p which induces apoptosis of β‐cell (Lee et al. 2023). Another EVs‐mediated protective pathway from β‐cells has been reported in the context of type 2 diabetes. Patients with type 2 diabetes frequently develop toxic amyloid deposits within their pancreatic islets, driven by the local buildup of islet amyloid polypeptide. EVs released by β‐cells from healthy individuals can suppress amyloid formation in the islets, whereas EVs derived from individuals with type 2 diabetes lack this protective effect. Investigators propose a novel mechanism to maintain islet homeostasis in physiological conditions. This self‐protection mechanism involves EVs proteins and lipids that are altered in pathological conditions, preventing its role in homeostasis maintenance (Ribeiro et al. 2017).

##### 3.1.4.2 Reproductive Endocrine System

EVs also play essential roles in reproductive endocrine health in females. Follicular fluid contains EVs released by mural granulosa cells and cumulus cells and has been shown to influence follicular growth and their content changes throughout follicular maturation (Machtinger et al. 2021; Navakanitworakul et al. 2016). The EVs of small follicles are enriched with pro‐proliferative miRNAs, while the EVs of mature follicles contain pro‐inflammatory miRNAs. In particular, EV miR‐204, miR‐92b, and miR‐328a‐3p, miR‐424‐3p, and miR‐450a were upregulated when comparing mature follicles with small follicles (Navakanitworakul et al. 2016). During fertilization, EVs containing CD9 are released from egg cells, which interact with sperm cells to facilitate sperm‐egg fusion (Miyado et al. 2008). After fertilization, the cell surface receptor Juno, which facilitates sperm‐oocyte fusion, is rapidly shed in EVs to prevent sperm cell fusion with the fertilized oocyte (Bianchi et al. 2014). In two separate studies in humans, oocytes that failed to fertilize had a different EV miRNA profile than those that normally fertilized. Machtinger et al. (2017) identified miR‐202‐5p, miR‐206, miR‐16‐1‐3p, and miR‐1244; Martinez et al. (2018) identified miR‐766‐3p, miR‐663b, miR‐132‐3p, miR‐16‐5p, miR‐888, miR‐214, and miR‐454. These studies suggest that EV miRNAs can be used to assess successful fertilization and viability.

EVs have been implicated in various reproductive pathologies, such as polycystic ovary syndrome (PCOS), endometriosis, preeclampsia (PE), and infertility. Multiple studies have detected altered miRNA profiles in follicular fluid‐derived EVs, suggesting that dysregulated EV signaling contributes to disrupted folliculogenesis, steroidogenesis, and metabolic‐reproductive crosstalk. EVs from endometrial or immune cells may promote lesion establishment and maintenance by modulating inflammation, angiogenesis, and local steroid receptor signaling in the peritoneal microenvironment; reviewed in (Çine et al. 2025; Duval et al. 2024). In polycystic ovary syndrome (PCOS), several studies show that follicular fluid–derived EVs carry altered miRNA profiles. For example, miR‐424‐5p was found to be significantly decreased in EVs from PCOS follicular fluid, and EV‐encapsulated miR‐424‐5p inhibited granulosa cell proliferation and induced senescence by targeting CDCA4 via the Rb/E2F1 pathway (Yuan et al. 2021). In preeclampsia (PE), placenta‐derived miRNAs carried by EVs have been profiled: in a preliminary study of maternal plasma EVs, differential expression of 7 miRNAs was found in preeclampsia versus controls. These miRNAs may illuminate disease‐relevant pathways or contribute to the diagnosis of preeclampsia. EV‐encapsulated miR‐31‐5p (from placenta and peripheral blood) was found to be significantly downregulated in PE, and its levels correlated (negatively) with clinical indicators like hypertension and proteinuria, suggesting use as a biomarker (Zou et al. 2022).

In males, EVs (especially from Sertoli cells, testicular endothelial cells, and the reproductive tract) contribute to spermatogenesis, testicular microenvironment, and hormonal regulation. Sertoli cell‐derived EVs inhibit apoptosis of primary spermatogonia via delivery of miR‐10b, which targets KLF4 (Gao et al. 2023). In another line, Sertoli cell EVs could promote differentiation of spermatogonial stem cells (SSCs): miR‐486‐5p enriched in EVs derived from Sertoli cells downregulates PTEN, and upregulates Stra8 in SSCs, facilitating their differentiation (Gao et al. 2023). Testicular endothelial cell‐derived EVs (TEC‐EVs) have been profiled by proteomics and miRNA arrays: in human TEC‐EVs, 945 proteins and 2578 miRNAs were identified, with 11 proteins linked to spermatogenesis and 30 miRNAs associated with male reproductive disorders, implying that these EVs may modulate germ cell–endothelial communication (Song et al. 2021).

### 3.2 Interorgan Communication

In addition to cell‐to‐cell interaction, EVs have been identified as critical contributors to long‐distance communication between different organs (Zhang and Grizzle 2014). Long‐distance EV‐mediated communication contributes to organism development. Verweij et al. employed CD63‐pHluorin, a fluorescent reporter to facilitate live visualization of EV release in single cells (Verweij et al. 2019). This reporter is specifically targeted to endosomes and then secreted on EVs and tracked during zebrafish development, demonstrating the release of EVs from the yolk syncytial layer into circulation. These EVs are specifically taken up by macrophages and endothelial cells of the caudal vein plexus to be degraded in acidic endo‐lysosomes. Interference with EV biogenesis affected the growth of the caudal vein plexus. The authors concluded that endothelial cells may uptake and degrade EVs to use them as a resource of molecules for their own development.

Over the past several years, studies aimed to identify the mechanisms behind EV‐mediated interorgan communication and the clinical application of EVs have faced several challenges, including clarifying biodistribution. The mechanisms for EV biodistribution are an emerging field of study. The cell of origin, route of administration, and EV composition are all important factors that contribute to EV biodistribution (Gurung et al. 2021; Morishita et al. 2017; Murphy et al. 2019; Munagala et al. 2016). Different mechanisms of EV uptake have been reported to be dependent on the recipient cell (Horibe et al. 2018). For example, rabies viral glycoprotein (RVG), which interacts with the acetylcholine receptor, has been shown to enable EV delivery to the brain (Cui et al. 2019; Alvarez‐Erviti et al. 2011). Alvarez‐Erviti et al. used dendritic cell‐derived EVs engineered to express LAMP2B, fused to the neuron‐specific RVG peptide. Once intravenously injected, these EVs targeted specifically neurons, microglia, and oligodendrocytes in the brain, without non‐specific uptake of EVs by other tissues (Alvarez‐Erviti et al. 2011). Laulagnier and colleagues found that amyloid precursor protein (APP) and its C‐terminal fragments (CTFs) are sorted into EVs. Specifically, neuroblastoma cells release CD63‐positive EVs that target neuronal dendrites, while CD63‐negative EVs target both neurons and glial cells (Laulagnier et al. 2018) and could be partially responsible for the intercellular transport of APP and its catabolites in the brain. Organotropism of EVs has also been identified in cancer metastasis, with integrins α6β4 and α6β1 associated with lung metastasis, while integrin αvβ5 targeted liver metastasis (Hoshino et al. 2015). New methods of molecular imaging have been developed to track the biodistribution of EVs in the system. These methods include fluorescent, nuclear, bioluminescence, magnetic resonance, computed tomography, photoacoustic, and multimodal imaging, extensively described by Petroni et al. (2023). These methodologies have expanded our understanding of intercellular and interorgan signaling networks. However, the biodistribution of EVs can be affected by dosage, route of administration, and labeling process (Petroni et al. 2023). Moreover, EVs may undergo several cycles of cell uptake and release to access several layers of tissues (Gurung et al. 2021), which makes it more difficult to track the route of EVs from the cell of origin to the final destination.

**FIGURE 3:**
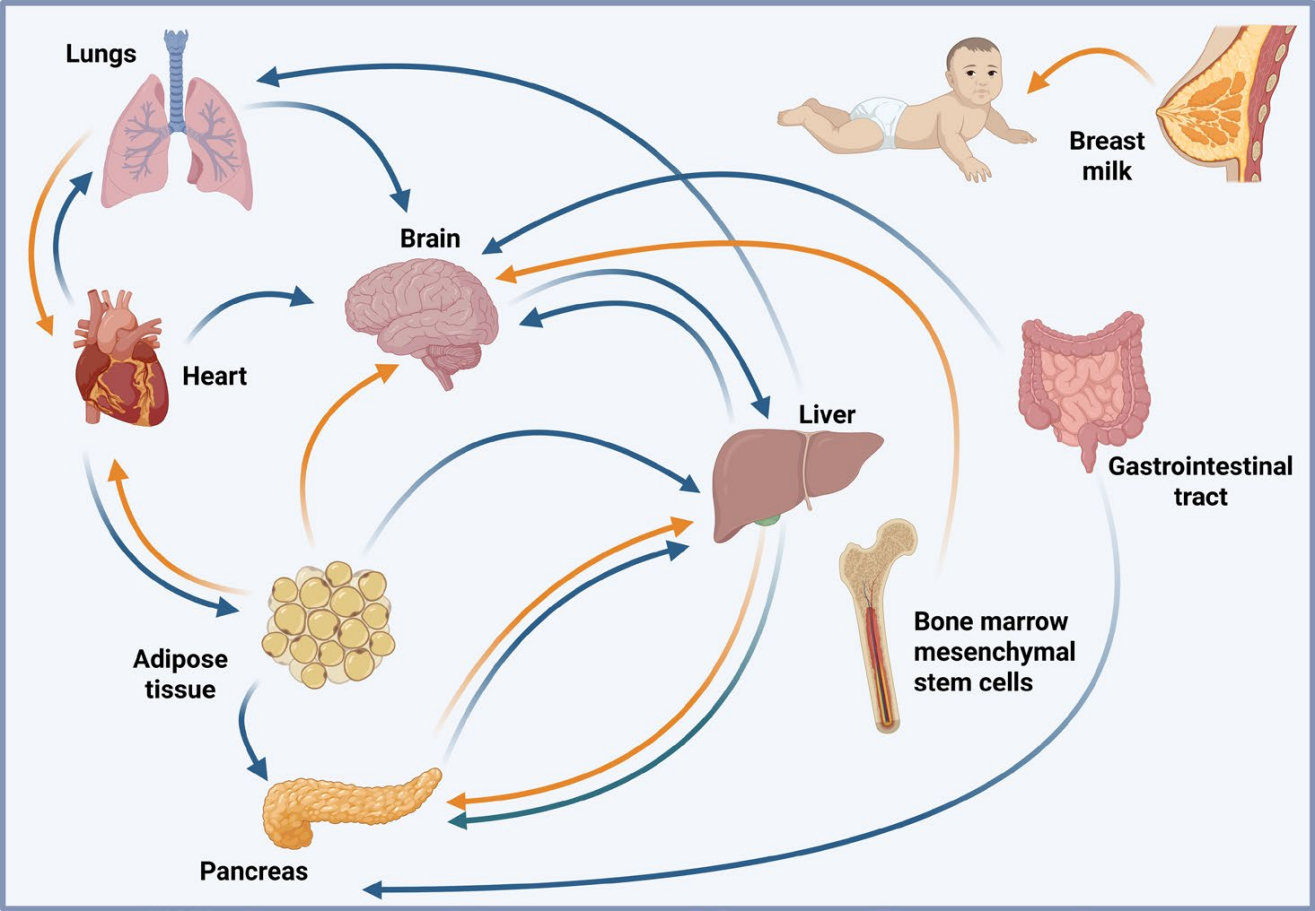
Representation of extracellular vesicles‐mediated interorgan communication. EVs secreted from the cells in both physiological and pathological conditions can travel throughout the body and potentially reach every distal organ. The figure highlights the direction of the EVs released from specific organs and directed to distant organs. The color of the arrows indicates beneficial (yellow) or pathological (blue) EVs communication mentioned in the main test. Created in https://BioRender.com.

Despite the difficulties of determining the origin of EVs, an increased number of studies have expanded our knowledge on the bidirectional communication between different organs through EVs, highlighting critical contributions of vesicles to both physiological and pathological conditions. In this section, we provide an overview of the latest research in the field of EVs as mediators of interorgan crosstalk. We provide an overview of the EVs secreted by several organs and how these EVs interact with other organs (summarized in Figure 3). EV‐encapsulated miRNAs involved in these processes are summarized in Table 1.

#### 3.2.1 Milk‐Derived EVs

EVs represent an interesting conduit for communication between mother and infant through breastfeeding. It has been shown that breast milk‐derived EVs (BMDEVs) play an important role in infant development. Colostrum and mature human BMDEVs contain MHC class II and the tetraspanin proteins CD63 and CD81. Interestingly, PBMCs incubated with milk EVs can increase the number of Foxp3^+^CD4^+^CD25^+^ T regulatory cells and inhibit anti‐CD3‐induced cytokine production, suggesting a potential role of maternal EVs in driving infant immune response (Admyre et al. 2007). Several lines of evidence suggest that BMDEVs contribute to the maturation of the neurological, gastrointestinal, and immune systems of the infant (Papakonstantinou et al. 2024; Alsaweed et al. 2015; Zeng et al. 2020). Kim et al. demonstrated that BMDEVs actively promote the differentiation of naïve CD4+ T cells, enhancing the development of Tregs and Th2 subsets, while suppressing the differentiation into pro‐inflammatory Th1 and Th17. BMDEVs also attenuate the production of pro‐inflammatory cytokines and inflammatory mediators in macrophages and alleviate the severity of colitis in vivo (Kim et al. 2025). In vitro and in vivo studies on necrotizing enterocolitis prevention demonstrated the effectiveness of BMDEVs in preventing cell death and stimulating intestinal cell proliferation (Martin et al. 2018; Wang, Yan, et al. 2019). Human milk‐derived EVs can be internalized by intestinal stem cells, reducing the effect of oxidative stress in vitro (Dong et al. 2020). Milk‐derived EVs (MDEVs) from different species can mitigate necrotizing enterocolitis, promote intestinal epithelial cell growth, and alleviate LPS‐induced inflammation and cell apoptosis (Muttiah and Law 2025). Several studies have indeed reported that these EVs attenuate the expression of inflammatory cytokines such as IL‐6, TNFα, NF‐KB, and IL‐1β (Vahkal et al. 2025).

Studies have shown that EVs from bovine milk can resist the process of digestion and are assimilated by intestinal cells, thus being available systemically (Kahn et al. 2018; Wolf et al. 2015; Mutai et al. 2020; Rani et al. 2017). As a result, MDEVs protect their RNA and protein cargos from RNases and proteinases (Rani et al. 2017). Manca and colleagues observed an accumulation of MDEVs in mice and pigs' livers, spleens, and brains after suckling, oral gavage, and intravenous administration. Interestingly, a synthetic fluorophore‐labeled miRNA contained in these EVs had a different distribution, accumulating mainly in the intestinal mucosa, spleen, liver, heart, or brain. The authors speculate that MDEVs are taken up and processed, and that the cargo miRNA is successively transferred out through endogenous EVs (Manca et al. 2018). In vitro and in vivo studies show how MDEVs can undergo uptake by human vascular endothelial cells by endocytosis (Kusuma et al. 2016), thus becoming available for transport to peripheral tissues. The consumption of milk helps maintain certain physiological functions, such as immunoregulation, maintenance of intestinal tract, muscles and bones (Zhong et al. 2023; Zeng et al. 2019).

#### 3.2.2 Bone Marrow‐Derived EVs

Vesicles from mesenchymal stem cells (MSCs) harbor protective and repair properties in the brain (Turano et al. 2023). Factors contained in EVs of bone marrow mesenchymal stem cells (BMSC) are found to have positive effects on the brain following injury. Liu et al. isolated EVs from primary BMSCs obtained from the femora and tibia of rats. Tail vein injection of these EVs can reduce the brain infarct area following cerebral ischemia–reperfusion injury and co‐culture experiments show that BMSC‐derived EVs modulate microglial polarization from M1 to M2 phenotype, suggesting that these EVs ameliorate cerebral injury by modulating microglial polarization (Liu et al. 2021). EVs derived from BMSCs protect against ischemic brain injury. Red fluorescent PKH26‐labeled EVs were injected into the tail vein in t‐MCAO mice and found in the brain tissue. MiR‐124 carried in these EVs seems to ameliorate stroke‐mediated injury and reduces proinflammatory microglia activation (Tian, Yao, et al. 2022). miR‐23a‐3p derived from BMSC EVs remedies cerebral infarction by decreasing the activation of microglia and favoring its polarization to the M2 phenotype with potential for treatment of cerebral infarction (Dong et al. 2022). MSC from bone marrow are a valuable source of EVs and promote neurite outgrowth in cortical neurons and in sensory neurons (Lopez‐Verrilli et al. 2016).

#### 3.2.3 Gut‐Derived EVs

A strong relationship between gut and brain is mediated by EV communication (Zhou, Zhao, et al. 2023). Gut‐derived vesicles can originate from both intestinal cells and microorganisms. Although gram‐negative bacteria‐derived vesicles are produced by budding the outer membrane, these particles can still penetrate the blood–brain barrier (BBB) and are reported to disrupt its integrity (Pirolli et al. 2021) and induce microglia and astrocyte activation, causing inflammation and cognitive impairment (Pirolli et al. 2021; Wei et al. 2020). EVs from intestinal cells can have beneficial effects on the brain (Inotsuka et al. 2021). However, it is important to consider that toxins or microbial infections can impact EVs produced from the intestinal cells and can subsequently affect the brain. Gao et al. found that bacterial DNAs are enriched in β‐cells of individuals with obesity, while is barely present in healthy subjects. Intravenous injection of PKH26‐labeled EVs derived from gut microbiota of obese subjects can transfer microbial DNAs to pancreatic β‐cells, impairing glucose‐stimulated insulin secretion and pro‐inflammatory gene expression by triggering cGAS/STING activation (Gao et al. 2022).

#### 3.2.4 Brain‐Derived EVs

The function of EV in the peripheral nervous system in interorgan communication has been extensively studied in health and disease (Bischoff et al. 2022). EVs can cross the blood–brain barrier (Abdelsalam et al. 2023) and potentially reach every organ. Also, EV populations derived from mice, human, cancerous, and noncancerous cell lines can bidirectionally cross the BBB at different rates (Banks et al. 2020). A growing amount of evidence reports bidirectional communication between the brain and different organs, such as the liver, lung, gastrointestinal tract, bone marrow, and adipose tissue, through EVs (Zhou, Zhao, et al. 2023). In the nervous system, EVs from Schwann cells are involved in the maintenance of homeostasis and regeneration of peripheral nerves (Bischoff et al. 2022; Lopez‐Verrilli et al. 2013). EVs not only modulate on‐site response in the nervous system but can also be transported systemically throughout the body. Using a mouse model of inflammatory brain injury via injection of interleukin‐1β, Dickens et al. demonstrated that astrocyte‐derived EVs from GFAP‐GFP mice rapidly cross the BBB and target peripheral organs. GFP‐positive EVs were found enriched in the liver and, to confirm that EVs originate from the brain, the authors labeled brain cells with FM 1–43. In the liver, astrocyte‐shed EVs modulate PPARα and stimulate the secretion of inflammatory cytokines, which support leukocyte priming and translocation to the brain, providing a new insight into the regulation of immunological response after brain damage (Dickens et al. 2017).

#### 3.2.5 Liver‐Derived EVs

The ApoE4 allele is a major risk factor for Alzheimer's Disease (AD). Peripheral ApoE4 is produced mainly in the liver, but the link between APOE4 and cognitive impairment and metabolism is unclear. Zhang et al. found increased levels of APOE4 in serum EVs from old subjects and AD patients compared to healthy subjects, and an inverse correlation of serum levels of APOE4 with thyroid hormones and cognitive function. In vivo experiments have shown that EVs containing APOE4 secreted from the liver can be delivered to the brain in the context of aging‐associated hypothyroidism (Zhang et al. 2022). By injecting a lenti‐Arg1‐APOE4‐eGFP vector in the hepatic portal vein of mice, the authors were able to visualize the immunoreactivity of APOE4 that originated from the liver in the hippocampus and cortex of old and thyroidectomized animals, but not in adult healthy animals. Once in the brain, APOE4 activates the NLRP3 inflammasome by increasing neuron cholesterol levels, leading to cognitive impairment, revealing a new potential therapeutic target for APOE4‐associated AD and cognitive impairment. Miotto et al. recently reported that liver‐derived EVs are released in circulation in response to hyperglycemia. In vivo injection of liver‐derived EVs exhibits a response from the pancreas and skeletal muscle to increase insulin secretion and effectiveness of glucose, thus improving body glycemic control. This research highlights possible therapeutic implications of liver‐derived EVs in conditions of impaired glucose metabolism (Miotto et al. 2024). EVs have been implicated in both protective and pathogenic roles in type 2 diabetes and metabolic syndrome, with hepatocyte‐derived EVs containing miRNAs that modulate the systemic insulin response. In individuals with early‐onset obesity, hepatocytes often produce EVs that express high levels of the insulin‐sensitizing miR‐3075 (Ji et al. 2021). In contrast, in chronic obesity, hepatocyte‐derived EVs from chronic obese mice promote insulin resistance. Pancreatic Min6 cells, treated with EVs isolated from liver HepG2 cells under insulin resistance conditions, increased insulin gene expression and AKT signaling, suggesting a feedback communication from the liver to the pancreas (Mahmoudi‐Aznaveh et al. 2023). The relationship between the liver and lung can be observed in hepatopulmonary syndrome (HPS). Serum EVs from HPS rats induce pulmonary microvascular endothelial cells proliferation, migration, and tube formation (Chen et al. 2019). Bidirectional communication between the liver and other organs has been extensively reviewed by Mo et al. (2025).

#### 3.2.6 Adipose Tissue‐Derived EVs

Adipose‐derived stem cells have been established to promote cerebral vascular remodeling following stroke. In vitro studies showed that EVs derived from subcutaneous adipose tissue‐derived stem cells promote angiogenesis of brain microvascular endothelial cells through the miR‐181b‐5p/TRPM7 axis (Yang et al. 2018). By using EVs labeled with the red fluorescent dye, DiI, and injected into the tail vein, Yang et al. show that EVs from hypoxic, pre‐treated adipose‐derived stem cells ameliorate cognitive function in mice with cerebral infarction. These EVs decreased neuronal damage in the hippocampus via delivery of circ‐Rps5 and promoting polarization of the microglia to the M2 phenotype (Yang et al. 2022). EVs from adipose tissue also communicate with the liver. EVs released from brown adipocytes express high levels of miR‐99b, which can reach the liver, where they inhibit FGF21 expression (Thomou et al. 2017). EVs from adipose tissue impair insulin sensitivity and induce insulin resistance (Kita et al. 2019; Kranendonk et al. 2014) playing an important role in glucose intolerance (Huang and Xu 2021). Kulaj et al. demonstrate that adipocyte‐derived extracellular vesicles are taken up by pancreatic β‐cells. Here the protein cargo undergoes phosphorylation and activates insulinotropic GPCR‐cAMP‐PKA signaling enhancing glucose‐stimulated insulin secretion (Kulaj et al. 2023). Such an effect is limited to EVs derived from obese and insulin resistant mice, but not from lean mice, suggesting a physiologically adaptive role for adipocyte‐derived EV signaling during insulin resistance. Obese adipose tissue‐derived EVs contain low levels of miR‐141‐3p compared to healthy adipose tissue. EVs lacking in miR‐141‐3p are absorbed by hepatocytes and can significantly inhibit insulin sensitivity and glucose uptake (Dang et al. 2019).

EVs from adipose tissue communicate with the heart. In obesity, adipocytes are subject to energetic stress that results in loss of mitochondrial function (Crewe et al. 2021). Crewe and colleagues found that, under these conditions, mitochondrial stress induces adipocytes to secrete EVs containing oxidatively damaged mitochondrial particles. By labeling EVs with fluorescent dye PHK26, the authors show that cardiomyocytes can take up these EVs, which induce transient mitochondrial dysfunction and free radical production. The authors explain that this is not a pathological process but rather an adaptive physiological response protecting cardiomyocytes from acute oxidative stress (Crewe et al. 2021).

#### 3.2.7 Heart‐Derived EVs

Cardiac dysfunction is present in patients following neurologic injury and stroke, with 11%–18% of stroke patients suffering symptomatic heart failure (Chen et al. 2017). Otero‐Ortega et al. profiled both protein and miRNA contents in EVs, finding similarities between EVs from patients with myocardial infarction and patients with ischemic stroke compared to healthy controls (Otero‐Ortega et al. 2020). Using labeled cardiac‐derived EVs and cardiac‐specific membrane GFP^+^ transgenic mice, Tian et al. demonstrated that cardiac EVs were transported in the bloodstream and taken up by neurons in the rostral ventrolateral medulla (Tian, Gao, et al. 2022). A study from the Cortes group reported a downregulation of miR‐26a in EVs from urine and plasma of patients with hypertension with albuminuria, a marker of cardiovascular risk and renal damage in hypertension. miR‐26a is a regulator of TGF‐β signaling, and decreased levels of miR‐26a were found in podocyte‐derived EVs after TGF‐β‐induced stress (Martinez‐Arroyo et al. 2025).

Pironti and colleagues investigated EVs containing the angiotensin II type I receptor from the serum of mice undergoing cardiac pressure overload. These EVs are mainly secreted by cardiomyocytes, and the administration of EVs enriched in Angiotensin II type I transports them to skeletal muscle, cardiomyocytes, and mesenteric resistance vessels. In vivo, these EVs were shown to increase systolic blood pressure when injected into Angiotensin II type I KP mice (Pironti et al. 2015).

Bidirectional communication between heart and adipose tissue has been reported. Gao et al. isolated and labeled EVs derived from a myocardial infarction mouse model, which were internalized by adipose‐derived MSCs in vitro, enhancing their proliferation by activating ERK1/2. The organ patterns of EV secretion after myocardial infarction showed that these EVs are most likely released from the heart and kidney, and they might serve as messengers for stem cell regulation (Gao et al. 2020). EV‐mediated communication between injured heart and adipose tissue contributes to metabolic disorder. Gan et al. treated adipocytes with EVs isolated from cardiomyocytes subjected to in vivo myocardial ischemia/reperfusion. These EVs carry miR‐23‐27‐24 cluster which targets ER degradation enhancing alpha‐mannosidase like protein 3 which downregulation results in adipocytes' endoplasmic reticulum stress (Gan et al. 2022).

Recently, EVs have emerged as mediators of the relationship between heart failure and cancer (Koelwyn et al. 2022; Avraham et al. 2020; Meijers et al. 2018). A recent study investigated the link between post‐myocardial infarction (MI) left ventricular dysfunction and lung cancer. Researchers discovered that EVs secreted from cardiac mesenchymal stroma cells in the condition of post‐MI contain cytokines such as TGF‐β, TNF‐α, IL‐6, VEGF, and miRNAs (miR‐221, miR‐21, miR‐24, and miR‐214) that accelerate tumor growth in mice and activate pro‐tumorigenic macrophages (Caller et al. 2024).

#### 3.2.8 Pancreas‐Derived EVs

As previously mentioned, EV‐mediated intercellular communication between pancreatic cells is responsible for glucose homeostasis and insulin sensitivity. Nevertheless, long‐distance communication is also necessary in these physiological processes. In response to high blood levels of glucose, pancreatic β‐cells secrete EVs enriched in miR‐223 that are delivered to the liver and skeletal muscles to facilitate glucose uptake and maintain glucose homeostasis (Wei et al. 2023; Zhang et al. 2018). Another innovative study demonstrated that high levels of free fatty acids stimulate the secretion of miR‐29 s into EVs under physiological (fasting) and pathological (high‐fat diet) conditions. Intravenous administration of miR‐29s‐enriched EVs causes impaired insulin sensitivity. The authors show that pancreatic β‐cells‐derived miR‐29s can be taken up by the liver and modulate insulin sensitivity, while the disruption of miR‐29s in pancreatic β‐cells improved sensitivity in mice fed with a high‐fat diet. The study also confirms the upregulation of plasma levels of EVs and EV‐free miR‐29s in obese patients compared to normal patients (Li et al. 2021).

Metabolic disorders impact normal communication processes. EV‐mediated long‐distance communication from pancreas to other organs serves as an initiator of insulin resistance and β‐cell failure associated with diabetes (Wei et al. 2023). In this context, β‐cell‐derived EVs can promote insulin resistance in other organs, such as liver; at the same time, EVs from other organs can drive β‐cell dysfunction and insulin resistance. Pancreatic islets express LncRNA Reg1cp (Gharib et al. 1993). Guo et al. identified a mutation of this LncRNA (Mut‐Reg1cp) associated with an elevated risk of type 2 diabetes. EVs enriched in Mut‐Reg1cp are secreted into circulation by pancreatic islets and transferred to muscle and liver, where they impair insulin sensitivity by inhibiting AdipoR1 translation and adiponectin signaling, revealing a new mechanism for type 2 diabetes (Guo et al. 2022). Downregulation of miR‐26a in EVs derived from the islet of obese mice induces hepatic insulin resistance, and its restoration improves insulin sensitivity and metabolic homeostasis in the liver, thus preventing metabolic damage (Xu et al. 2020). Furthermore, low‐density lipoprotein (LDL) hypercholesterolemia can disrupt communication between β‐cells and hepatocytes. Langerhans β‐cells treated with LDL decrease mTOR/p70S6Kα activation, impairing insulin signal in hepatocytes (Guevara‐Olaya et al. 2022).

#### 3.2.9 Lung‐Derived EVs

Recently, Ahmed and colleagues investigated the effect of EVs from COVID‐19‐infected lungs on the brain. The authors employed a complex bioinformatic framework that integrates gene expression profiles of the human brain, transcriptome datasets of SARS‐CoV‐2–infected lungs, and RNA profiles from lung EVs. In this study, the authors concluded that transcriptional factors in EVs, including BCL3, JUND, MXD1, IRF2, IRF9, and STAT1, may contribute to neurodegeneration by impacting the gene regulatory pathway (Ahmed et al. 2021).

Lung‐to‐heart EV translocation has also been reported. A recent study demonstrated that nicotine inhalation during e‐cigarette/vaping promotes cardio toxicity. Mechanistically, lungs from mice exposed to nicotine secrete EVs enriched in ERK5. The lung‐isolated EVs were labeled and intramyocardially injected, showing uptake from the cardiomyocytes, where activated GATA4 and promoted pyroptosis, uncovering a new role of lung EVs in exacerbating cardiotoxicity during vaping (Liu et al. 2024).

## 4 Conclusion

Studies on the role of EVs in intercellular and long‐distance communication have made great strides in recent decades and are contributing to our understanding of various pathological processes. Production, secretion, transport, and internalization of EVs are refined processes and highly susceptible to any small perturbation of the cellular environment. Participating in a complex communication system, EVs are particles enriched in molecular information regarding the status of the cells and the organ of origin. Furthermore, they can be transported in biological fluids and serve as biomarkers and inform pathological processes (Hanjani et al. 2022; Mosquera‐Heredia et al. 2021). EV content has been shown to impact cell signaling pathways in sepsis and contribute to organ injury (Murao et al. 2020). EV‐mediated signaling is crucial for metabolic diseases (Guay and Regazzi 2017), with EVs secreted by pancreatic β‐cells that regulate insulin sensitivity and immune responses in target tissues. Additionally, EVs derived from cancer cells can secrete factors that promote metastasis formation in distal organs (Luo et al. 2023; Steinbichler et al. 2017).

From a clinical perspective, EVs generated in vitro could serve as vehicles for organ‐targeted therapies. Li and colleagues developed an intriguing clinical application for EVs called Stem Cell‐derived Exosome Nebulization Therapy (SCENT) (Li, Sun, et al. 2024). EVs isolated from lung spheroid cells were delivered by a nebulizer to distal lungs to promote cardiac repair following myocardial infarction. The researchers observed the passage of EVs from the lung to the infarcted myocardium in 60 min, with EV signal reduction in the lung consistent with increased signal in the heart. In mouse models, daily SCENT treatment ameliorated cardiac repair by improving left ventricular function and reducing fibrotic tissue. An initial explanation of this result is the enrichment of miR‐100 in EVs, which downregulates CD63 in endothelial cells, thus promoting neovascularization and protecting cardiac tissues. Given the proximity of the lungs to the heart, this route is ideal for targeting ischemic tissue without side effects in other organs. Such a noninvasive delivery strategy of beneficial EV represents an important step forward in the clinical application of EV. Targeting EVs for treating diseases is an important focus of pharmacological research. EVs can be engineered by modifying the surface or content (Li, Qi, et al. 2024). Therapeutic strategies, such as nanoparticles or siRNAs, could be designed to prevent the binding of EVs to target organs or downregulate the expression of EV cargo. In cancer therapeutics, integrins like αvβ5 on the EV membrane can be blocked with monoclonal antibodies or engineered for targeted delivery of therapeutic molecules (reviewed in Ferreira, Moreira, and Rodrigues 2022).

The potential pharmacological regulation of EVs‐mediated communication is undeniable. Further, the concept of intercepting EV‐mediated signaling as a means for treating multi‐organ disease is intriguing but has yet to come to fruition. However, more studies are required to implement our knowledge of the mechanisms and regulation of EV‐mediated communication, as well as improved standardization, which is essential for data reproducibility. The identification of donor and recipient organs, as well as the evaluation of the biological impact of circulating EVs on the distant organ in vivo, represents a challenge that requires the establishment of specific methods and animal models. Nevertheless, in the past decade we have witnessed a rapid increase in new methodologies that open the field of EV biology to new precision therapies and personalized medicine.

## Author Contributions

Michela Saviana, Giulia Romano, and Daniel del Valle‐Morales prepared figures and drafted the manuscript; Mario Acunzo revised the manuscript, and Patrick Nana‐Sinkam edited and revised the manuscript. All the authors approved the final version of the manuscript.

## Funding

National Cancer Institutes 5UG1CA287017‐02 (to P.N.‐S.), National Center for Advancement of Translational Science 5K12TR004364‐02 (to P.N.‐S.) National Institutes of Health (NIH 1R21CA277525‐01) (to M.A.), American Lung Association (LCDA‐922902) (to M.A.).

## Disclosure

The authors have nothing to report.

## Conflicts of Interest

The authors declare no conflicts of interest.

## Data Availability

Data sharing not applicable to this article as no datasets were generated or analysed during the current study.
